# From meniscal resection to meniscal repair: a journey of the last decade

**DOI:** 10.1007/s00167-020-06316-7

**Published:** 2020-10-19

**Authors:** Roland Becker, Sebastian Kopf, Romain Seil, Michael T. Hirschmann, Philippe Beaufils, Jon Karlsson

**Affiliations:** 1 Centre of Orthopaedics and Traumatology, Brandenburg Medical School Theodor Fontane, Hochstrasse 29, 14770 Brandenburg an der Havel, Germany; 2 Department of Orthopaedic Surgery, Centre Hospitalier de Luxembourg-Clinique d’Eich, 76, rue d’Eich, 146 Luxembourg, Luxembourg; 3 Department of Orthopaedic Surgery and Traumatology, Kantonsspital Baselland (Bruderholz, Liestal, Laufen), 4101 Bruderholz, Switzerland; 4 Orthopaedic Department, Centre Hospitalier de Versailles, 177, rue de Versailles, 78157 Le Chesnay, France; 5 Department of Orthopaedics, Sahlgrenska University Hospital, Sahlgrenska Academy, University of Gothenburg, Gothenburg, Sweden


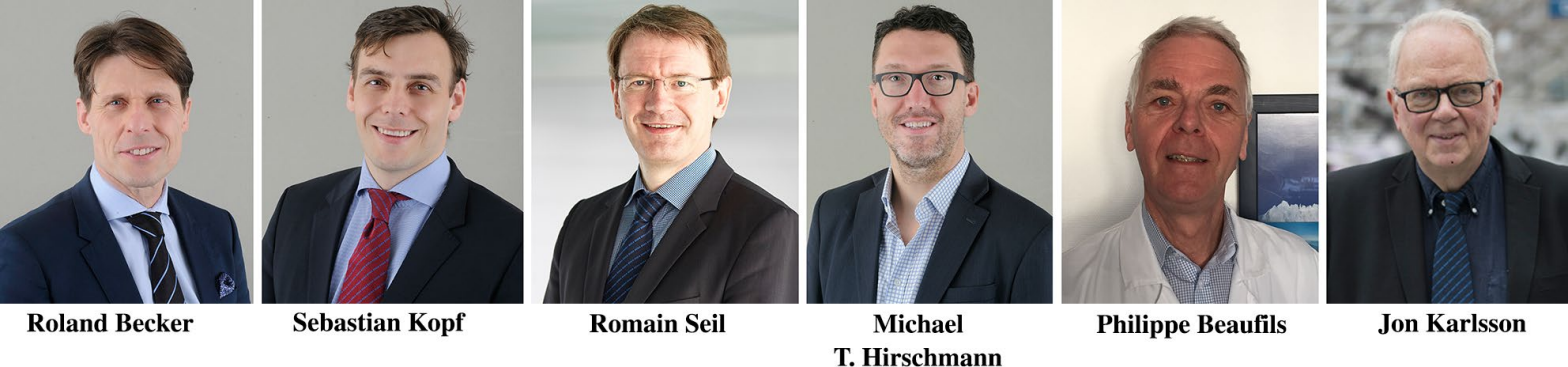
The last decade has shed some light on the darkness surrounding the treatment of meniscal injuries. A significant amount of work has been done in order to provide a more scientific approach to the treatment of the injured meniscus.

Degenerative meniscal lesions and traumatic meniscal tears differ in terms of aetiology and pathology and require differentiated diagnostic algorithms and treatments. A new terminology has; therefore, been defined by the ESSKA meniscus consensus project. A traumatic meniscal tear is caused by an acute and sufficiently serious trauma to the knee. In contrast, a degenerative meniscal lesion occurs due to repetitive minor injuries and lacks a sufficiently serious single trauma. The second European Consensus has studied the epidemiology, diagnosis and treatment of traumatic meniscal tears [14]. It follows the first consensus on the management of degenerative meniscal lesions, which was published in 2017 [3]. Both consensus reports combined basic science and knowledge of the clinical experience of more than 80 knee experts throughout Europe [3, 14, 23].

There are major differences in terms of the management of acute traumatic meniscal tears and degenerative meniscal lesions. While magnetic resonance imaging (MRI) should be performed early in traumatic tears for a satisfactory assessment of the pathology, there is no need for an immediate MRI when a degenerative meniscal lesion is suspected. An MRI will not only provide information about the location, type and size of the meniscal tear but also about the cartilage and ligament integrity, which is important for correct surgical planning.

Complete meniscal resection was the primary treatment option for any type of meniscal tear in the past. The orthopaedic mindset has changed markedly over the last decade. The importance of the meniscus in terms of shock absorption, knee stability, load distribution, lubrication, proprioception and neuromuscular function has been well recognised over the years [1, 4, 17]. Based on these findings, there is general agreement about preserving the meniscus whenever possible [21, 22]. Studies have shown that the preservation of traumatic meniscal tears does in fact reduce the risk of early osteoarthritis [18, 26]. Scientific evidence and a better understanding of meniscal pathology on the one hand and improvement in the surgical technique on the other have shifted the pendulum towards meniscal preservation. New suture devices enable fast, safe and easy techniques for handling meniscal repair using an all-inside technique when compared with outside-in or inside-out techniques and have become the standard for many surgeons. In the same way, a behavioural change with respect to meniscal treatment has occurred among many orthopaedic surgeons. In France, meniscal resection has decreased by 21.4%, while there was a threefold increase in meniscal repairs between 2005 and 2017 [10]. The reduction in meniscal resection was especially apparent in patients below 40 years of age. The meniscus repair ratio also increased in Japan from 9 to 25% between 2011 and 2016 [13]. This might be a direct result of the improved understanding of meniscal pathologies.

Acute meniscal tears are more frequent than previously thought. These tears are mainly longitudinal in nature, including bucket handle tears or radial and some types of root tear. They are often specifically associated with ligament injuries. Our awareness and understanding of these specific injury types have increased. Meniscal pathologies such as ramp lesions or root tears, for instance, have a high incidence and have recently attracted more interest. It has been reported that ramp lesions occur in up to 25% of anterior cruciate ligament (ACL) ruptures, with a higher incidence in contact injuries when compared with non-contact injuries [24, 25]. Ramp lesions are a good example when it comes to illustrating the increased awareness of meniscal injuries. This type of lesion is often missed when only the standard anteromedial and anterolateral portals are used [27]. In fact, most of them only become apparent if a notch view or an additional posteromedial portal is used to probe the meniscus. Increased anterior translation or delay in anterior cruciate ligament surgery are the main factors causing ramp lesions [27]. Likewise, lateral posterior root tears are also common in conjunction with ACL injuries. Although they are more easily recognised than ramp lesions, they were mostly neglected in the past. During the last decade, a novel description of specific classification systems and the development of new fixation techniques for these lesions were introduced [15].

Meniscal repair is a clinically successful procedure in more than 85% of patients; however, not all the repaired menisci heal completely [2, 12, 19]. These data show that there is still a need for a further understanding of meniscal anatomy, biology and healing [28]. This will remain an important field of research over the coming years. Initial reports on the use of platelet-rich plasma (PRP) or more specific combinations of growth factors or stem cells have revealed promising results in improving meniscal healing, especially in the avascular zone [11, 20]. However, the risk of misusing biological treatments in a poorly regulated environment is real and we are therefore obliged to be very careful with general recommendations with regard to these techniques.

In the past, meniscal repair was predominantly recommended for younger patients. This has changed and nowadays the patients’ biological age is gaining momentum in the decision-making process of repair versus resection as opposed to the chronological age. A specific injury in middle-aged patients is a posterior root tear of the medial meniscus. This is common in this age group and its natural history has shown the rapid progression of osteoarthritis or the development of subchondral insufficiency fractures and osteonecrosis. Early reports on root repair have shown clinical improvement; however, meniscal extrusion was not reduced and the progression of osteoarthritis remains the subject of debate [8].

A recent study of a small group was able to show less osteoarthritic progression after posterior medial root repair in patients with an average age of 47 years and Kellgren and Lawrence stage II [6]. A literature review also reported a decrease in the incidence of osteoarthritis when medial meniscus root tears were repaired [9]. More research is definitively needed in this field in the near future before any final conclusion can be drawn.

More recently, meniscal repair has also been used for meniscal lesions of a degenerative nature. This is the case with horizontal and complex lesions which frequently extend into the meniscal periphery. In the past, the principles of arthroscopic surgery recommended resecting meniscal tissue until a stable peripheral rim was obtained. Today, new concepts, which aim only to remove the loose parts of the tear and stabilise the periphery by vertical suture repair, are emerging [7]. No difference in Lysholm or Knee Osteoarthritis Outcome Scores have been reported when comparing the repair of horizontal and longitudinal meniscal tears in 40-year-old patients after 35 months of follow-up [16].

Preserving not only the meniscal periphery but also as much meniscal tissue as possible, because of the importance in terms of femorotibial load transmission, sounds logical [5].

Hence, if arthroscopy is performed, how much meniscal tissue should be resected and how much of it is suitable for repair? As yet, this question cannot be answered and the concept of combined resection and repair needs to be further examined in large-scale clinical studies.

This editorial shows that the scope for meniscus repair is greater than before and there is still a need for both more basic science and clinical research in order to identify the best practice when treating different meniscal pathologies.
